# 3D Printing Bioceramic Porous Scaffolds with Good Mechanical Property and Cell Affinity

**DOI:** 10.1371/journal.pone.0143713

**Published:** 2015-11-30

**Authors:** Chih-Hao Chang, Chih-Yang Lin, Fwu-Hsing Liu, Mark Hung-Chih Chen, Chun-Pin Lin, Hong-Nerng Ho, Yunn-Shiuan Liao

**Affiliations:** 1 Department of Orthopedics, National Taiwan University Hospital and National Taiwan University College of Medicine, Taipei, Taiwan; 2 Department of Mechanical Engineering, National Taiwan University, Taipei, Taiwan; 3 Department of Mechanical Engineering, LungHwa University of Science and Technology, Taoyuan, Taiwan; 4 Department of Dentistry, School of Dentistry, National Taiwan University Hospital and National Taiwan University, Taipei, Taiwan; 5 Department of Obstetrics & Gynecology, National Taiwan University Hospital and National Taiwan University College of Medicine, Taipei, Taiwan; University of Umea, SWEDEN

## Abstract

Artificial bone grafting is widely used in current orthopedic surgery for bone defect problems. Unfortunately, surgeons remain unsatisfied with the current commercially available products. One of the major complaints is that these products cannot provide sufficient mechanical strength to support the human skeletal structure. In this study, we aimed to develop a bone scaffold with better mechanical property and good cell affinity by 3D printing (3DP) techniques. A self-developed 3D printer with laser-aided gelling (LAG) process was used to fabricate bioceramic scaffolds with inter-porous structures. To improve the mechanical property of the bioceramic parts after heating, CaCO_3_ was added to the silica ceramic slurry. CaCO_3_ was blended into a homogenous SiO_2_-sol dispersion at weight ratios varying from 0/100 to 5/95 to 9/91 (w/w). Bi-component CaCO_3_/SiO_2_-sol was prepared as a biocomposite for the 3DP scaffold. The well-mixed biocomposite was used to fabricate the bioceramic green part using the LAG method. The varied scaffolds were sintered at different temperatures ranging from 900 to 1500°C, and the mechanical property was subsequently analyzed. The scaffolds showed good property with the composite ratio of 5:95 CaCO_3_:SiO_2_ at a sintering temperature of 1300°C. The compressive strength was 47 MPa, and the porosity was 34%. The topography of the sintered 3DP bioceramic scaffold was examined by SEM, EDS and XRD. The silica bioceramic presented no cytotoxicity and good MG-63 osteoblast-like cell affinity, demonstrating good biocompatibility. Therefore, the new silica biocomposite is viable for fabricating 3DP bone bioceramics with improved mechanical property and good cell affinity.

## Introduction

The repair or replacement of injured or defective bone is a critical problem for orthopedic surgeons. Bone scaffolds are among the many alternatives for both autograft and allograft, which provides optimal osteo-conductivity and osteo-inductivity. These materials provide the benefit of avoiding unwanted immunological responses, and they eliminate the risk of acquiring infectious diseases (AIDS and hepatitis) from graft tissue and body fluid [1]. Therefore, bone scaffold, which is biomimetic in both structure and chemical factor coating, is usually used for bone surgery to repair defects [2,3]. The selection of materials for bone scaffolds must incorporate the consideration of issues such as mechanical properties and bonding strength at the scaffold-bone interface.

Traditional scaffold manufacturing methods include particulate leaching, freeze-drying, fiber bonding, phase separation and sponge soaking [4–6]. Using these methods, higher porosity can be achieved. The internal structure of the scaffold, such as the pore size, pore shape and interconnectivity of the 3D scaffold, is difficult to control [7,8]. Additive manufacturing (AM) technology, also called 3D printing (3DP), has emerged recently. The advantage of this method is the easy creation of specific shapes that normally cannot be produced using traditional methods. Therefore, many studies have used this technology to fabricate scaffolds, producing the required pore shape, pore size, surface morphology and scaffold shape [2,9–11].

Recently, silica bioceramics have been widely used for bone restoration and bone tissue engineering because they have good mechanical properties, biocompatibility and bioactivity [12]. In particular, CaSiO_3_ (wollastonite) has been extensively studied and used in medical materials, including artificial bone and dental implants [13]. CaSiO_3_ has good bioactivity, biocompatibility and degradability [14]. The silicate materials bond rapidly to both hard and soft tissues and enhance bone regeneration [15]. Liu et al. used atmospheric plasma spray (APS) to deposit CaSiO_3_ onto Ti-6Al-4V plates, and the specimens were soaked in simulated body fluid (SBF). The results show that CaSiO_3_ dissolves to SiO_2_ and CaO. Ca^2+^ reacts with HPO_4_^2-^ in the SBF to induce precipitation of the apatite [16]. All of these data indicate that CaSiO_3_ is not only harmless to the human body but is also degraded and absorbed by the human body.

Unfortunately, CaSiO_3_ is very difficult to cut in order to form shapes, pores and structures. Therefore, producing a CaSiO_3_ scaffold with a uniform pore size and structure, controllable porosity and proper mechanical strength remains a significant challenge [17]. Ideal bone scaffolds require a three-dimensional porous structure and enough mechanical strength to provide structural support during bone growth and remodeling [18]. Generally, the compressive strength of human bone is 100–230 MPa [19]; however, the compressive strength of the SiO_2_ scaffold is only 4.2 MPa, which is insufficient to support the bone structure [5]. In this study, we mimic the mechanism of formation of the earth’s mantle by adding CaCO_3_ powder to SiO_2_-sol as a slurry. The biocomposite is formed at the lower melting temperature of CaCO_3_/SiO_2_-sol. CaSiO_3_ is metamorphosed from the CaCO_3_-SiO_2_ slurry after heat treatment. The SiO_2_-sol mixed with CaCO_3_ powder is used to form the ceramic green part with an inter-porous structure using a laser-aided gelling (LAG) method on a self-developed 3D printer. These silica bioceramic scaffolds can improve the mechanical property after heat treatment.

## Materials and Methods

### Preparation of the 3DP biocomposite materials

The principal materials used in this study were SiO_2_ powder with an average particle size of 25 μm and SiO_2_ sol with a sol solid content of 40% and 50-nm-diameter nanoparticles. The viscosity of 40% sol was found suitable for operation, and it was selected as the binder. The SiO_2_ sol and SiO_2_ powder were mixed at a 20/80 ratio (w/w) to produce a SiO_2_ slurry, termed CS0. The SiO_2_ sol formed the network structure to fabricate the green part after the gelling reaction, and hence its solid content would affect the mechanical property of the green part. Silica powder played the role of a filler substance in the network structure and intensified the mechanical property. CaCO_3_ was another powder additive that served as a filler substance and solid content after sintering. The slurry would become too sticky to spread evenly on the platform when the CaCO_3_ content was higher than 10%. Therefore, CaCO_3_ powder was added separately to the SiO_2_ slurry at weight ratios of 5% and 9%, termed CS5 and CS9, respectively.

### Assembly of the 3DP machine and fabrication of scaffold

**Fig 1:**
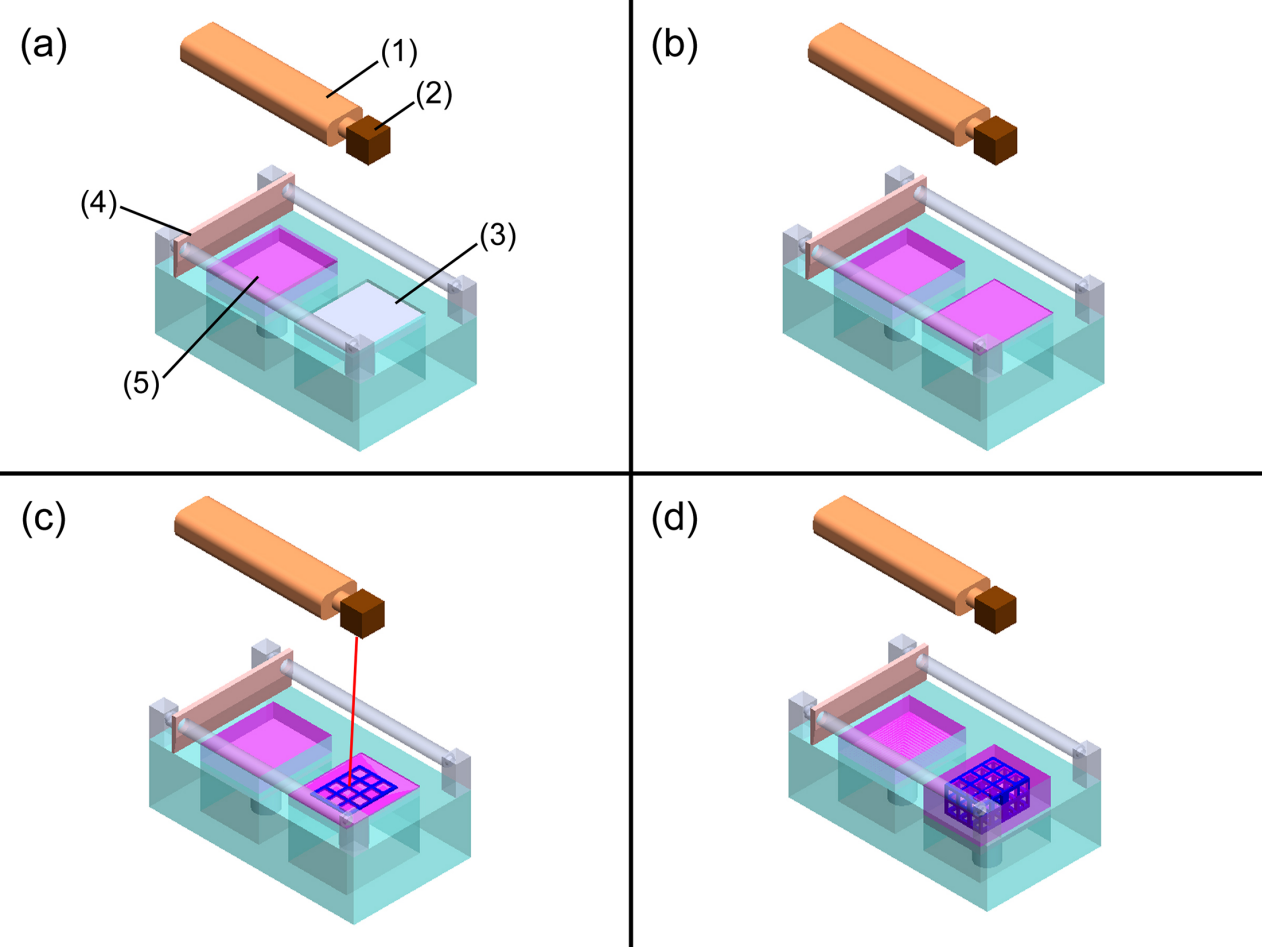
Schematics for the laser-aided gelling process. (1) a CO_2_ laser, (2) a laser scanner, (3) a working platform, (4) a scraper and (5) a feeder.

A home-made 3D printing machine was used, consisting of the following devices: (1) a CO_2_ laser, (2) a laser scanner, (3) a working platform, (4) a scraper, and (5) a feeder, as shown in Fig 1. The method used laser-aided gelling (LAG), as shown in Fig 1. The manufacturing process includes the following steps: (a) driving the servo motor to keep the feeding area at a fixed distance; (b) evenly paving the slurry in the feeding area on the surface of the working platform using a scraping plate and then returning the scraping plate to its original position after evenly paving the slurry in the forming area; (c) using the CO_2_ laser as the thermal energy source, scanning the shape for molding with the laser scanner until the moisture of the slurry evaporates and the shape sets; (d) steps (a) through (c) are repeated until the green part is finished. The slurry is removed by flushing with distilled water. This completes the production of the 3D green part. The curing reaction is irreversible, so the area that is not scanned in the slurry state is easily cleaned away by flushing with distilled water.

Specimens of Ø 6x12.5 mm dimensions and 4.5x4.5x1 mm dimensions were fabricated using LAG processes. The appropriate temperature in preparing calcium silicate had been studied, and it was found to range from 900 to 1400°C [20–23]. Therefore, heating of the green part specimens in a furnace (P310, Nabertherm, Germany) to a temperatures of 900°C, 1100°C, 1300°C and 1500°C was conducted in this study. The heating rate was 10°C/min, and the end temperature was maintained for two hours. The different samples of CS0, CS5, and CS9 were used to explore the improvement in mechanical property after sintering.

### Mechanical property analysis and characterization of the 3DP scaffold

Compressive strength was performed according to the JIS-R1608 standard. Cylindrical specimens of Ø 6x12.5 mm were used with a loading rate of 2 mm/min. Three samples were used to test the average compressive strength using a universal test machine (HT-9102, Hungta, Taiwan). The topography of the specimens and the crystal structure were observed using a scanning electron microscope (JSM-6500f, JEOL, Japan). The samples were prepared by mixing CaCO_3_ and SiO_2_ at ratios of 0/100, 5/95, and 9/91 (w/w). The oxygen (SiO_2_ powder, sol, CaCO_3_), Si (SiO_2_ powder, sol), and Ca (CaCO_3_) element distributions were examined by energy dispersive X-ray spectroscopy (EDS). The compositions of CS0, CS5 and CS9 were analyzed by X-ray diffraction (D/Max 2200, Rigaku, Japan) with CuK_α_. Each run was performed with 2θ values between 20 and 40 at a step of 5°/min. The porosities of the scaffolds were measured by the liquid displacement method [24]. The density and porosity were evaluated according to Archimedes’ principle. In these measures, all samples were tested at least in triplicate to obtain the average mechanical strength, density, volume expansion and porosity. The data are presented as the mean ± standard error of the mean (SEM) (n = 3–4 per group).

### Cytotoxicity test of the 3DP scaffold

The mammalian cell toxicity was measured according to the modified ISO method ISO 10993–5 for medical devices. The NCTC clone 929 (L-929) cell line was purchased from American Type Culture Collection (ATCC). All of the procedures were performed according to our previous research [25]. Cells were seeded in 24-well tissue culture plates at a density of 2 ×10^4^ cells per well. After 24 hours, the medium was removed and replaced with 0.1 mL of the sample to be tested (suspension of 3DP scaffold) and 0.9 mL medium. The cells were then incubated for another 24 hours. The negative control received no suspension but was still incubated for 24 hours at 37°C without material contact. All measurements were performed in three repeated wells as independent triplicates. After incubation at 37°C in 5% CO_2_ for 24 hours, cytotoxicity was measured, determining the metabolic activity of the cells by the tetrazolium salt test (MTT test). The optical density of the negative control was standardized at a wavelength of 570 nm at 100% and compared to the relative values of the test sample. The absorbance was measured at 570 nm using a microplate reader and normalized to the negative control to obtain cell viability.

### Cell Affinity of the 3DP scaffold

This study is focused on mechanical property, hence the cell affinity of the composite ratio of scaffold that led to the best compressive strength was tested. Human MG-63 cells were purchased from ATCC and used to test cell adhesion. The cells were directly suspended on the surfaces of ceramic disks in a 96-well plate with a density of 5000 cells/well. All measurements were performed in at least three repeated wells as independent triplicates. After incubation at 37°C in 5% CO_2_ for 24 hours, an MTT test was performed to determine the cell activity in the 3D scaffold. The MTT dye was converted by mitochondrial dehydrogenases, and the blue-purple formazan within the cells was quantified. The cell-ceramic scaffolds were washed three times with phosphate buffer solution and fixed with 2.5% (V/V) glutaraldehyde solution at 25°C. The morphology of bone cells on the surfaces of ceramic disks was observed by stereomicroscopy (SME-1500, Nikon, Japan) and imaged (Pro-150ES, Pixera, Japan). To observe cell details on the scaffold, we combined DAPI dihydrochloride (FluoroPure^™^ grade) and Alexa Fluor^®^ 488 phalloidin purchased from Invitrogen. The protocol was performed according to the manufacturer’s recommendation (Molecular probes, Invitrogen). DAPI represented the nuclear site, and phalloidin showed the actin cytoskeleton. Higher-resolution images were achieved by confocal microcopy (SP5, Leica, Germany). During the long-term attachment on the scaffold, the MG-63 cell medium was changed every 3 days until the last day of the assay. The scaffold was extracted from 96 wells, and an MTT assay was used to count the values. Measurements were obtained using a spectrophotometer (VersaMax, Molecular Devices, USA), and the readings were converted to cell numbers using a standard curve. The cell proliferation levels of MG-63 on the scaffold are presented as the mean plus standard error of the mean. Statistical analysis was performed by Student’s t-test on the values obtained from five independent wells. A P value < 0.01 was considered highly statistically significant.

## Results and Discussion

### Mechanical properties of bioceramic scaffolds

**Fig 2:**
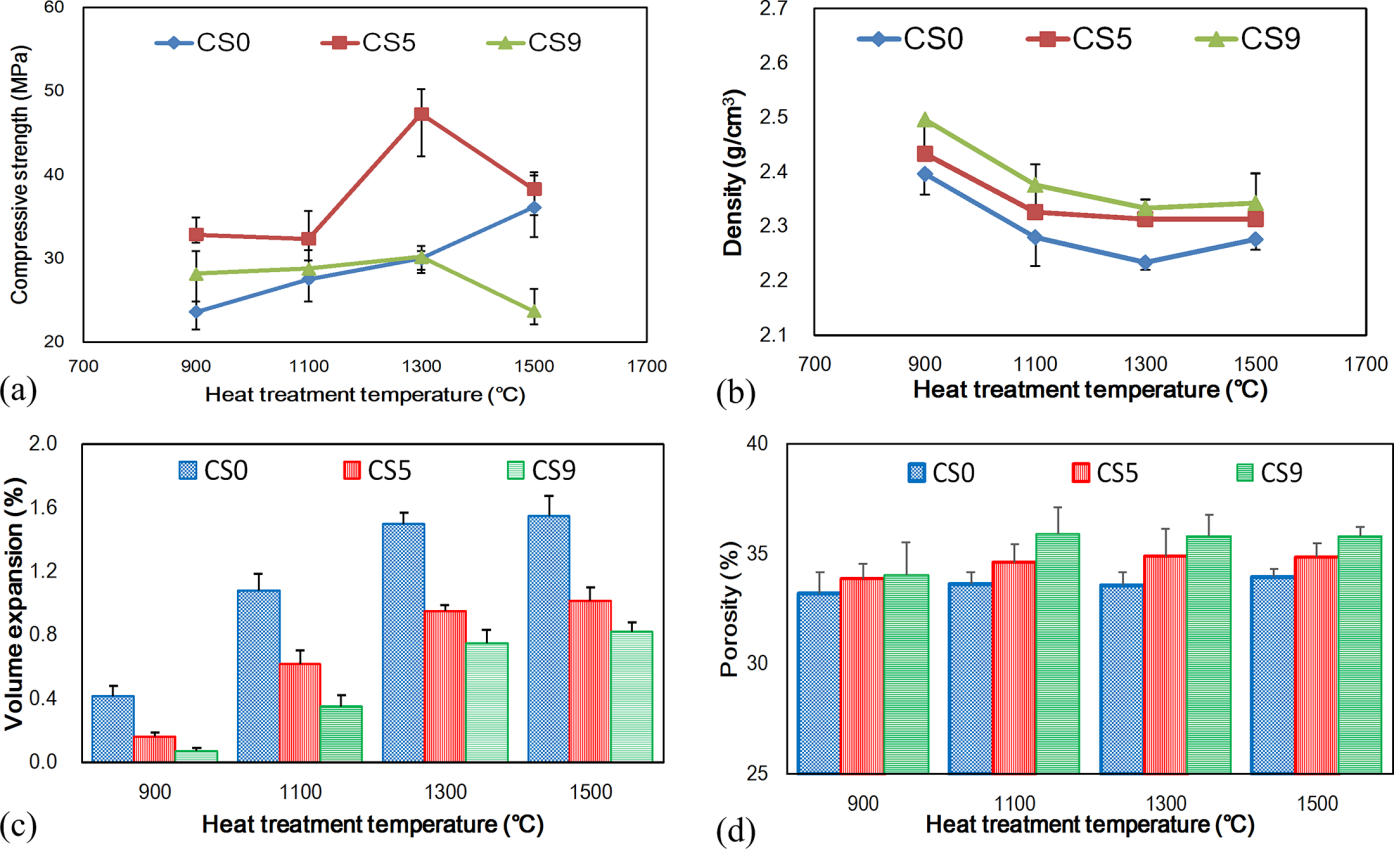
The compressive strength of specimens of CS0, CS5 and CS9 for different heat treatment temperatures:(a) compressive strength, (b) density, (c) volume expansion and (d) porosity.

Fig 2a shows the compressive strength of these specimens tested after heat treatment at different temperatures from 900–1500°C. It can be seen that the compressive strength of CS0 is improved with increasing temperature, and it is 36 MPa for the temperature of 1500°C. CS5 and CS9 demonstrated higher compressive strength than CS0 at 900°C, but their compressive strengths did not show any obvious change when the heat treatment temperature was increased from 900°C to 1100°C. At 1100°C, CS5 demonstrated higher compressive strength than CS0, but CS9 showed similar compressive strength to CS0. At 1300°C, the compressive strength of CS5 was obviously improved. The highest compressive strength is 47 MPa, which represents a 30% improvement over CS0 and CS9. The compressive strength of CS9 shows no obvious improvement at 1300°C. When the temperature of the heat treatment was increased to 1500°C, the strengths of both CS5 and CS9 were dramatically decreased. The compressive strength of CS9 was lower than the compressive strength of CS0, whereas the compressive strength of CS5 was only 2 MPa higher than CS0 at 1500°C. The above data show that the mechanical property of the bone scaffold is clearly improved not only by the addition of CaCO_3_ but also by increasing the sintering temperature.

The change of the compressive strength with respect to temperature is explained as follows. The initial temperature in this study was 900°C because the reaction of CaCO_3_ and the SiO_2_ sol began at 900°C. At this temperature point, the compressive strength of CS5 and CS9 were higher than that of CS0. This is because that CaCO_3_ starts to react with SiO_2_ sol to precipitate calcium silica crystal at 900°C. At 1100°C, the calcium silica crystal is still at growth procedure, so CS5 and CS9 do not change in compressive strength. For CS0, because the recrystallization of the SiO_2_ sol is proportional to the increase in temperature, the compressive strength increases gradually, as shown in Fig 2a. At 1300°C, the compressive strength of CS5 is higher than 900°C and 1100°C, the reason is calcium silicate has been completely grown [23].

The melting point is another factor that affects the mechanical strength in the sintering procedure. The compressive strength of CS0 still increased with temperature gradually because the temperature did not reach the melting point of SiO_2_ (1650°C). From 1300–1500°C, the compressive strength of CS5 and CS9 decreased because the melting point of calcium silicate is 1540°C. The 1500°C sintering temperature is thus very close to the melting point. With increasing temperature, more calcium silicate melts. As more calcium silicate melts, the layer covering the SiO_2_ powder surface becomes thinner. Thus, the compressive strength of CS5 and CS9 decreased in the 1300–1500°C range.

### Density, volume expansion and porosity of bioceramic scaffolds

The densities of CS0, CS5, and CS9 at different temperatures are shown in Fig 2b. It was can be seen that the density increased in proportion to the CaCO_3_ concentration regardless of the temperature. Upon increasing the sintering temperature, the density decreased due to volume expansion in all samples (Fig 2b and 2c). The silica crystal phase changed after 1100°C, as it has been previously demonstrated that silica transforms from quartz to cristobalite after heating [26]. The densities of quartz and cristobalite are 2.5 g/cm^3^ and 2.3 g/cm^3^, respectively. Thus, the volume expansion of scaffolds increased gradually with temperature until 1300°C. This phenomenon corresponds to the volume expansion, as shown in Fig 2c. The porosities of CS0, CS5, and CS9 are shown in Fig 2d. The porosity of CS0 is lower than in CS5 and CS9 regardless of sintering temperature. The CS5 and CS9 porosities increased slightly from 33% to 34% and 34% to 35% in proportion to the temperature increase from 900°C to 1100°C, respectively, because CO_2_ evaporates from the CaCO_3_ additive after sintering. The porosity was stable after sintering at temperatures from 1100°C to 1500°C because CaCO_3_ reacted completely, and no CO_2_ was produced, as shown in Fig 2d. Though CS5 has a greater porosity than CS0, it has the better mechanical property.

### Surface topography of bioceramic scaffolds

**Fig 3:**
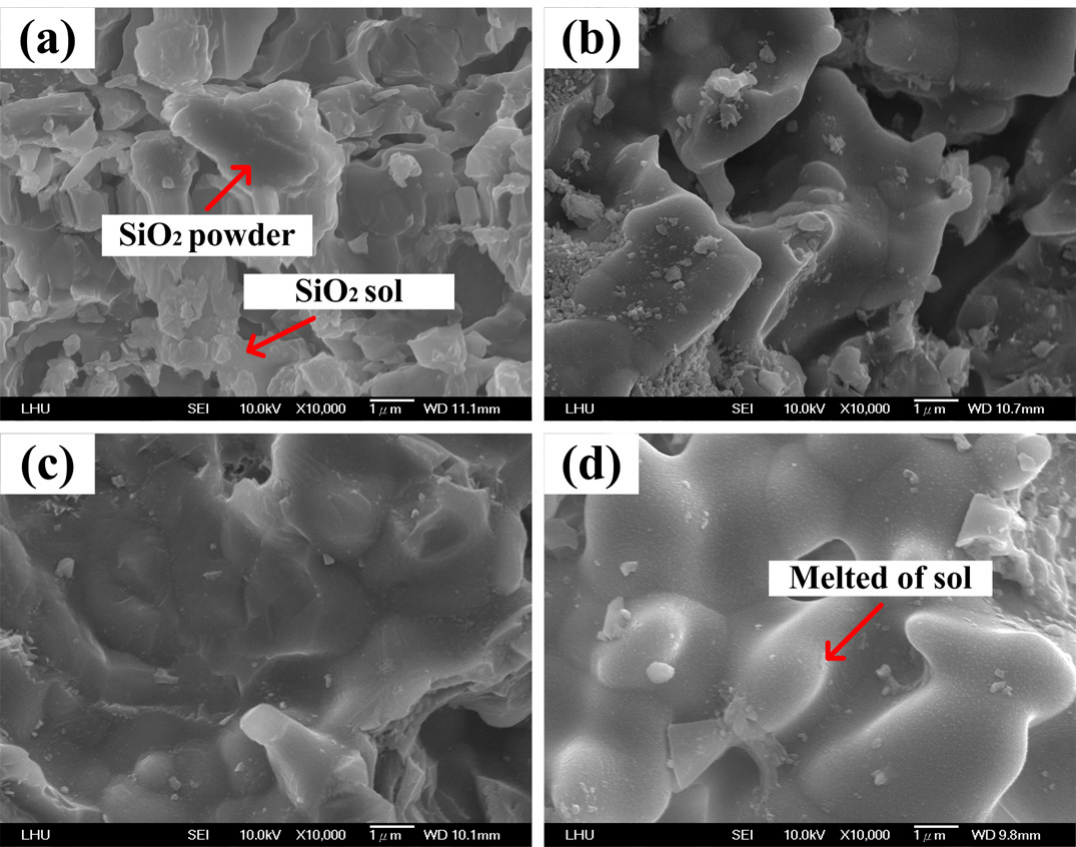
The microstructure of CS0 after heat treatment at various temperatures:(a) 900°C, (b) 1100°C, (c) 1300°C and (d) 1500°C.

To better understand why CS5 presented the best mechanical strength at 1300°C, the surface topographies of specimens CS0, CS5 and CS9 were examined using SEM. When the heat treatment temperature for CS0 is set at 900°C, part of the SiO_2_ sol gradually recrystallizes and fills the pore space, covering the surface with massive SiO_2_ crystals, as shown in Fig 3a. At 1100°C, the SiO_2_ crystals on the surface of CS0 begin to agglomerate and SiO_2_ sol coats the SiO_2_ powder as shown in Fig 3b. At 1300°C, most of the SiO_2_ sol on the surface of CS0 agglomerates into larger pieces, as shown in Fig 3c. At 1500°C, the surface of CS0 appears to be a massive smooth structure and all of the SiO_2_ powder is coated by SiO_2_ sol, as shown in Fig 3d. The structure is denser than the structures produced at the other three heat treatment temperatures, resulting in a higher compressive strength. The compressive strength gradually increases along with the bonding strength of the SiO_2_ sol, as shown in Fig 2a.

**Fig 4:**
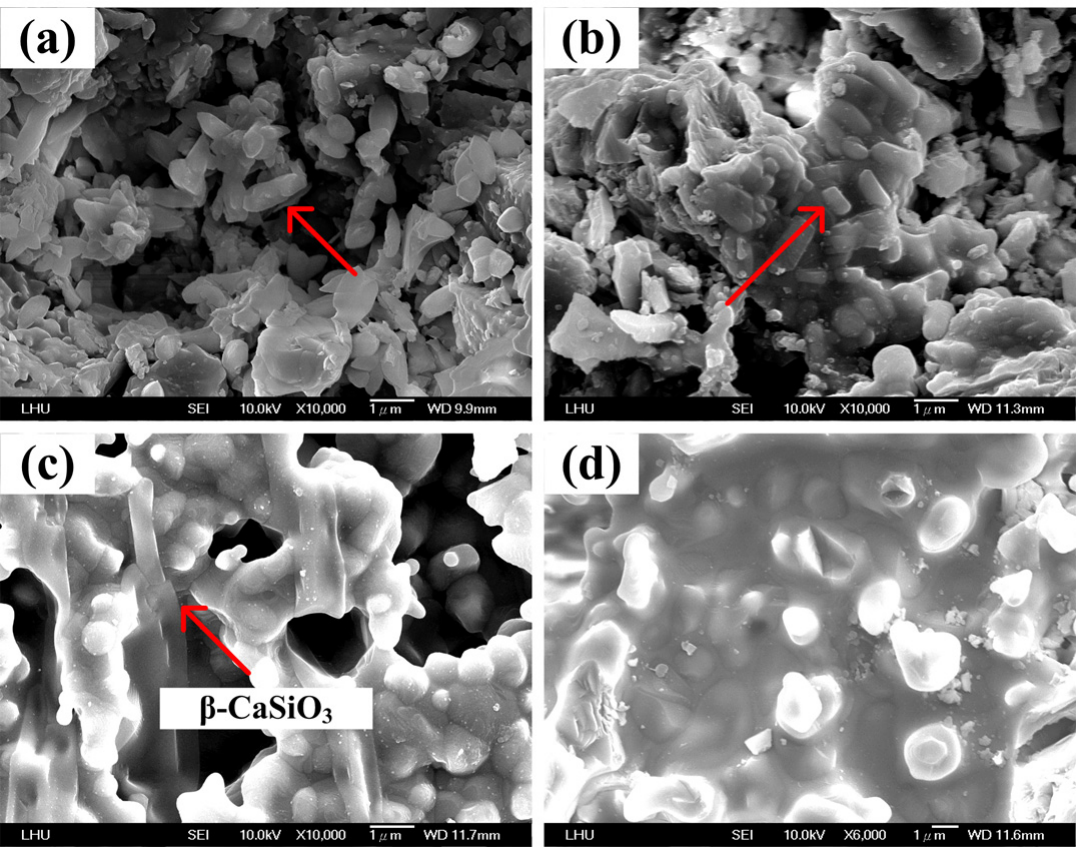
The microstructure of CS5 after heat treatment at various temperatures:(a) 900°C, (b) 1100°C, (c) 1300°C and (d) 1500°C.

When the heat treatment temperature for CS5 is 900°C, the surface separates out CaSiO_3_ if CaCO_3_ is added and, as observed in Fig 4a, the surface is evenly covered with rhombic-like crystals with a size of approximately 1 μm. When the temperature is set to 1100°C, the surface begins to grow needle-like crystals characteristic, as shown in Fig 4b. Long noted that needle-like crystal was β-CaSiO_3_ [27]. When the heat treatment temperature is 1300°C, β-CaSiO_3_ crystals are also observed on the CS5 surface, and the needle-like crystals are larger than those at 1100°C. The needle-like crystals have a lattice structure and can resist stress rupture, resulting in a significant improvement in compressive strength, as shown in Figs 2a and 4c. When the temperature is 1500°C, the needle-like crystals disappear and oval-like crystals characteristic of α-CaSiO_3_ begin to precipitate, as shown in Fig 4d. It has been previously demonstrated that oval-like crystal is α-CaSiO_3_ [28].

**Fig 5:**
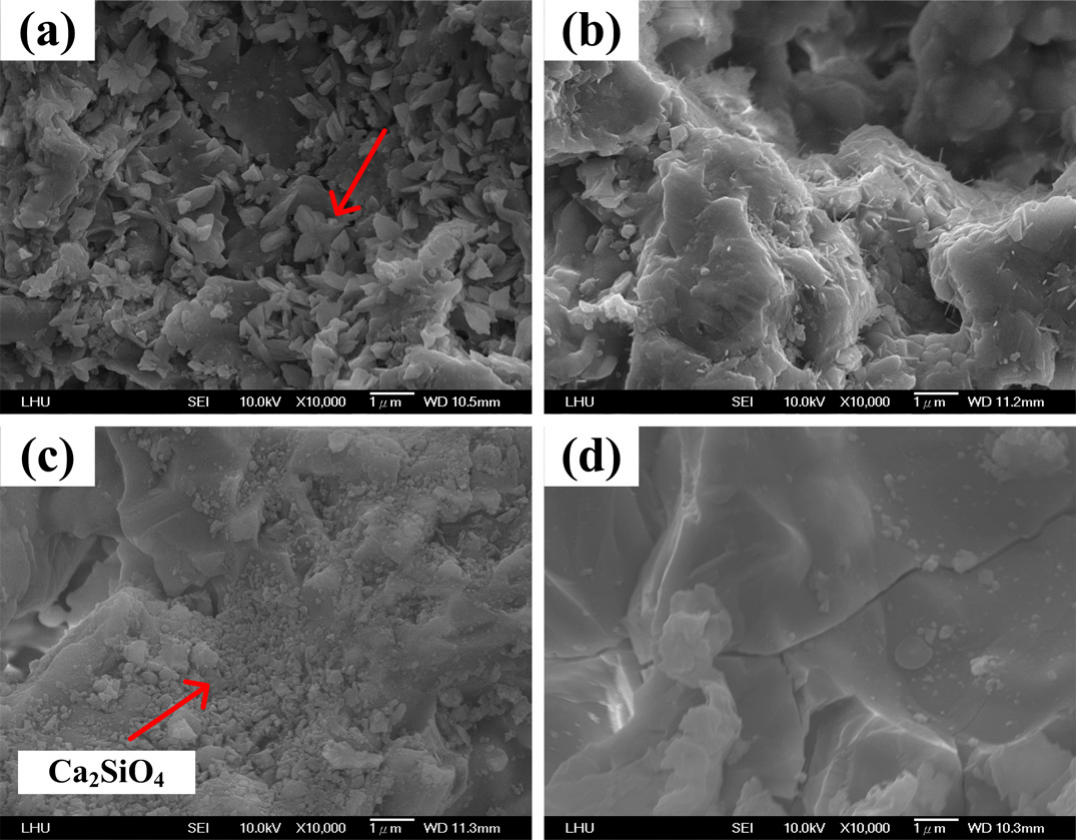
The microstructure of CS9 after heat treatment at various temperatures:(a) 900°C, (b) 1100°C, (c) 1300°C and (d) 1500°C.

When CS9 is heated to 900°C, the surface precipitates rhombic crystals, similar to CS5, and the crystal size is approximately 0.5 μm. This result demonstrates that an increase in the CaCO_3_ content results in a metamorphic state in the size of the rhombic-like crystals, as shown in Fig 5a. When the temperature is raised to 1100°C, the needle-like crystals of CS9 is smaller than CS5, and the CaCO_3_ content of CS9 is higher than CS5. Hence, the needle crystal may be Ca_2_SiO_4_ which has a lower compressive strength than CaSiO_3_ [29]. Our compressive strength results are shown in Fig 2a and indicate that the CS9 structure is poorer than the CS5 structure. When the temperature rises to 1300°C, the needle-like crystals on the surface of CS9 disappear, as shown in Fig 5c. When the temperature is 1500°C, a massive smooth structure is formed, but the surface begins to crack and the strength of the structure is weakened as shown in Fig 5d. This process results in a sudden decrease in the compressive strength (Fig 2a).

The surface results show that when CaCO_3_ is added and the heat treatment temperature is increased, the surface crystal structures are altered as observed by SEM examination, and the compressive strength values surge, as found using the testing machine. The heating experiments demonstrate that the temperature of 1300°C ensures the right crystal growth in CS5.

### Chemical composition of bioceramic scaffolds

**Fig 6:**
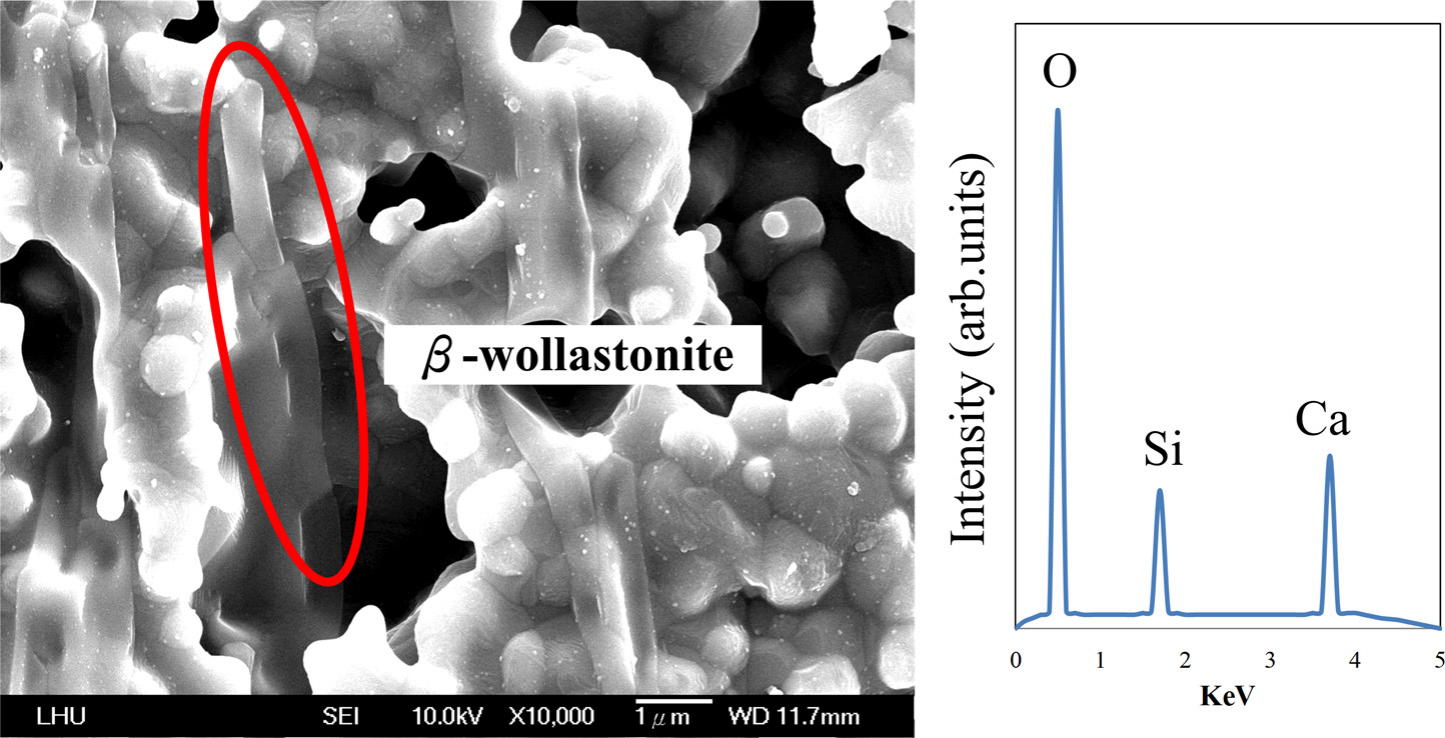
The EDS of CS5 after heat treatment at 1300°C.

The element analysis of needle-like structure of CS5 under 1300°C heat treatment temperature (i.e. marked area of Fig 4c) by EDS was conducted, and the result is shown in Fig 6. The composition of elements is Ca, Si and O, in proportions of approximately 1:1:3, resembling the elemental proportions of CaSiO_3_. In addition, the needle-like crystal morphology is characteristic of β-CaSiO_3_ crystals [30]. Hence, it is conducted that the crystal is β-CaSiO_3_. The EDS analysis results show that when the temperature increases from 1300°C to 1500°C, the element content and proportions of CS5 stay the same, but the crystal phase of CS5 may change from β-CaSiO_3_ to α-CaSiO_3_.

Regarding the chemical transformation of CS0, CS5, and CS9, we analyzed the melting temperature of SiO_2_ and found it to be very high (1650°C). At the melting point, the structure of SiO_2_ is ruined. To improve the mechanical properties and maintain the structure of SiO_2_, Pach et al. combined CaCO_3_ with SiO_2_ to form CaSiO_3_ [30]. In our blast-furnace results, CaCO_3_ underwent pyrolysis at 900°C, producing CaO and releasing CO_2_ gas. Furthermore, from SEM micrographs, it can be observed that the SiO_2_ powder retains a lump shape in CS0 (Fig 3). CaO reacts with SiO_2_ at all heat treatment temperatures. We propose that the SiO_2_ initially reacts with CaO in CS5 between 900 and 1300°C. The results in Fig 2a confirm that the mechanical properties are improved with the addition of the appropriate amount of CaCO_3_ and sintering at the optimum 1300°C. Otherwise, the compressive strength decreases if excess CaCO_3_ is added (as in CS9) because excess Ca may induce the formation of Ca_2_SiO_4_. The compressive strength of Ca_2_SiO_4_ is poorer than that of CaSiO_3_ [29], consistent with our results (Figs 2a and 5d). In summary, the appearance of α-CaSiO_3_ and Ca_2_SiO_4_ in the 3D scaffold due to heat treatment and calcium addition, respectively, result in poor mechanical property (Figs 4d and 5d). The precipitation and growth of β-CaSiO_3_ crystals require the optimum amount of CaCO_3_ and temperature of 1300°C.

**Fig 7:**
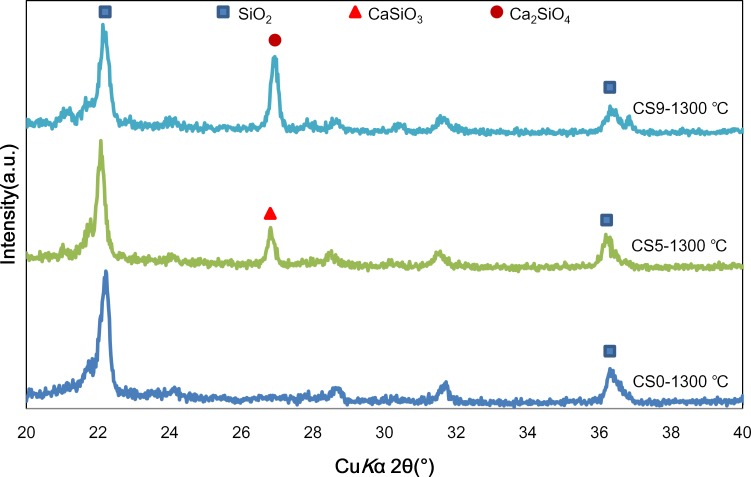
XRD of CS0, CS5 and CS9 after heat treatment at 1300°C.

Fig 7 shows the XRD data for CS0, CS5 and CS9 at 1300°C. In the CS0 curve, only the SiO_2_ peak appears. In the CS5 curve, there are two crystal phase peaks, SiO_2_ (PDF No.82–1560) and CaSiO_3_ (PDF No.84–0654). In the CS9 curve, there are two crystal phase peaks, SiO_2_ and Ca_2_SiO_4_ (PDF No.31–0302).

### Bioceramic scaffolds with inter-porous channel

**Fig 8:**
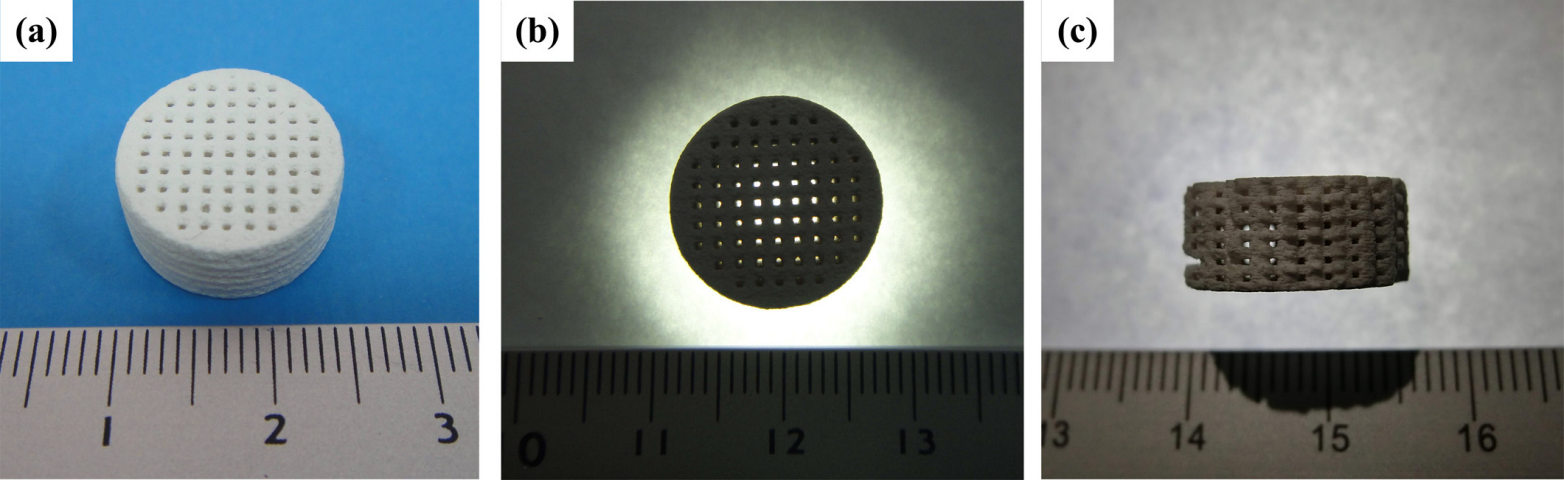
A 3D bioceramic part with inter-porous structure and dimensions of Ø 15 × 6.5 mm produced using laser-aided gelling:(a) isometric view, (b) top view and (c) cross section.

The optimal material prescription and heat treatment temperature were used to produce an inter-porous bioceramic model for use in bone scaffolds. The inter-porous bioceramic scaffold model, designed using the Solidworks software, has dimensions of Ø 15x6.5 mm, a pore size of 0.8x0.8 mm and a pore distance of 0.8 mm. The forming conditions were a laser scanning velocity of 100 mm/s, a laser power of 3.5 W, a scanning pitch of 0.1 mm, a laser frequency of 10 kHz and a thickness per layer of 0.1 mm, for 65 layers. Fig 8a shows the 3D bio-ceramic model produced by laser-aided gelling. The pores in the scaffolds were connected, as shown in Fig 8b and 8c shows an inner channel in horizontal section, demonstrating that all channels in the scaffold structure were connected, based on the light emitted from the porous parts. The use of LAG to form scaffolds allows allowed the pore shape, size and arrangement to be controlled.

The pores of the scaffolds are an important structure. Table 1 compares of the various fabrication methods used to form bone scaffolds [9,14,31–37]. The methods of foam replication, sponge soaking and 3DP are used to fabricate bone scaffold with materials such as bio-glass, CaSiO_3_, HA, tricalcium phosphate (TCP) and Al_2_O_3_. These methods are used with polymer as a binder because the binding of ceramics is poor. To form a pure ceramic scaffold, it is necessary to burn out the polymer by heat treatment, in which the residual volume is replaced by micropores. As shown in Table 1, the porosity remains high, in the range of 30–90%. However, the compressive strength of the scaffold is less than 20 MPa because the boundary melting of the supporting structure is accompanied by polymer burnout. In this study, we have successfully chosen novel biocomposites—CaCO_3_/SiO_2_ —to form porosity-strength CaSiO_3_ bioceramic scaffolds using LAG and 1300°C sintering as shown in Fig 8. Based on our design, the compressive strength and porosity of biomimetic human bone scaffold reached 47 Mpa and 34%, respectively.

**Table 1:** A comparison of the materials, methods and features that are used for the fabrication of scaffolds.

Reference	Materials	Porosity (%)	Compressive Strength (MPa)	Methods
[34]	Bioglass	79–89	0.1–1.4	Foam Replication
[9]	CaSiO_3_/SiO_2_/ZnO	89	0.14	Foam Replication
[36]	CaMgSi_3_O_6_	75–90	0.6–1.6	Sponge soaking
[14]	HA/CaSiO_3_	88–91	0.21–1.02	Sponge soaking
[31]	Al_2_O_3_/SiO_2_	56	18	Sponge soaking
[35]	Bioglass	60	16	3DP
[33]	SiO_2_/ZnO	32–52	2–10	3DP
[37]	α/β-TCP	41–53	0.9–8.7	3DP
[32]	β-TCP	45–55	2.3–8.7	3DP

### Cell affinity of the 3DP scaffold

**Fig 9:**
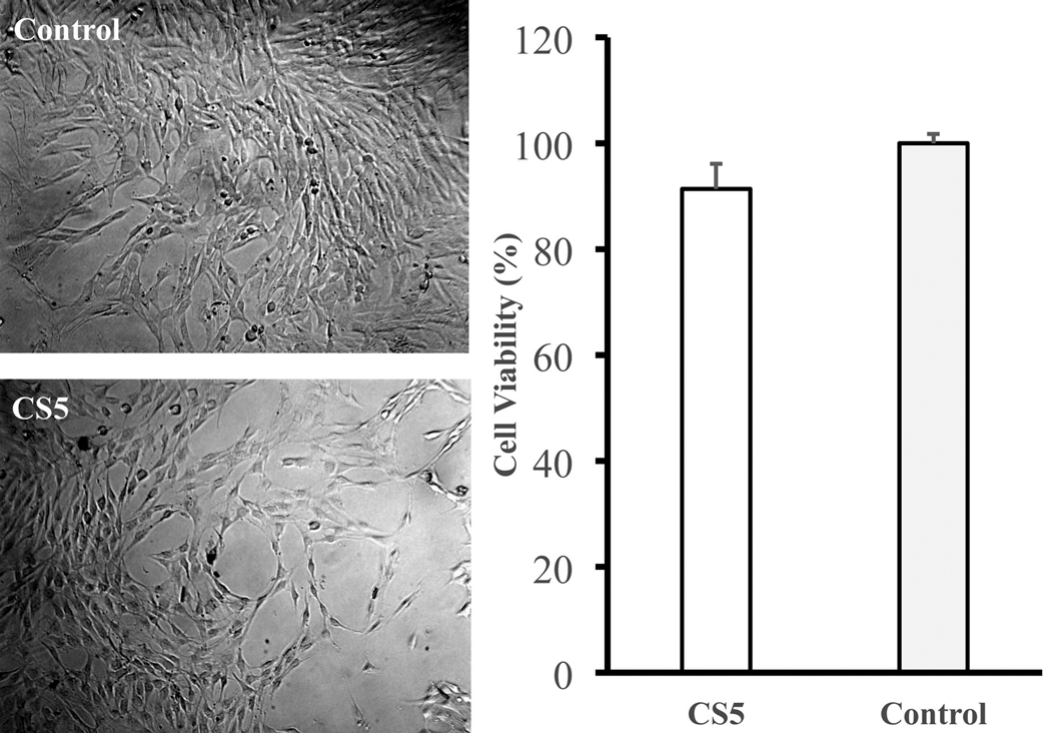
Mammalian cytotoxicity of the CS5 scaffold. Mammalian cell toxicity (or viability for L-929 cells) of the CaCO_3_ and SiO_2_ (5/95 by weight composition) scaffold indicated an appropriate safety level based on the ISO 10993–5 method.

After optimizing the strength and porosity of the bone scaffold in CS5, the next important issue is cell affinity to the artificial bone. We must determine whether this scaffold is non-toxic before it is implemented in clinical usage. For the safety test, the 3DP scaffold was shown to be nontoxic to mouse fibroblasts using ISO protocol 10993–5. The cytotoxicity test result of CS5 is 91%; according to the ISO-10993-5 criteria, this value indicates no cytotoxicity as shown in Fig 9.

**Fig 10:**
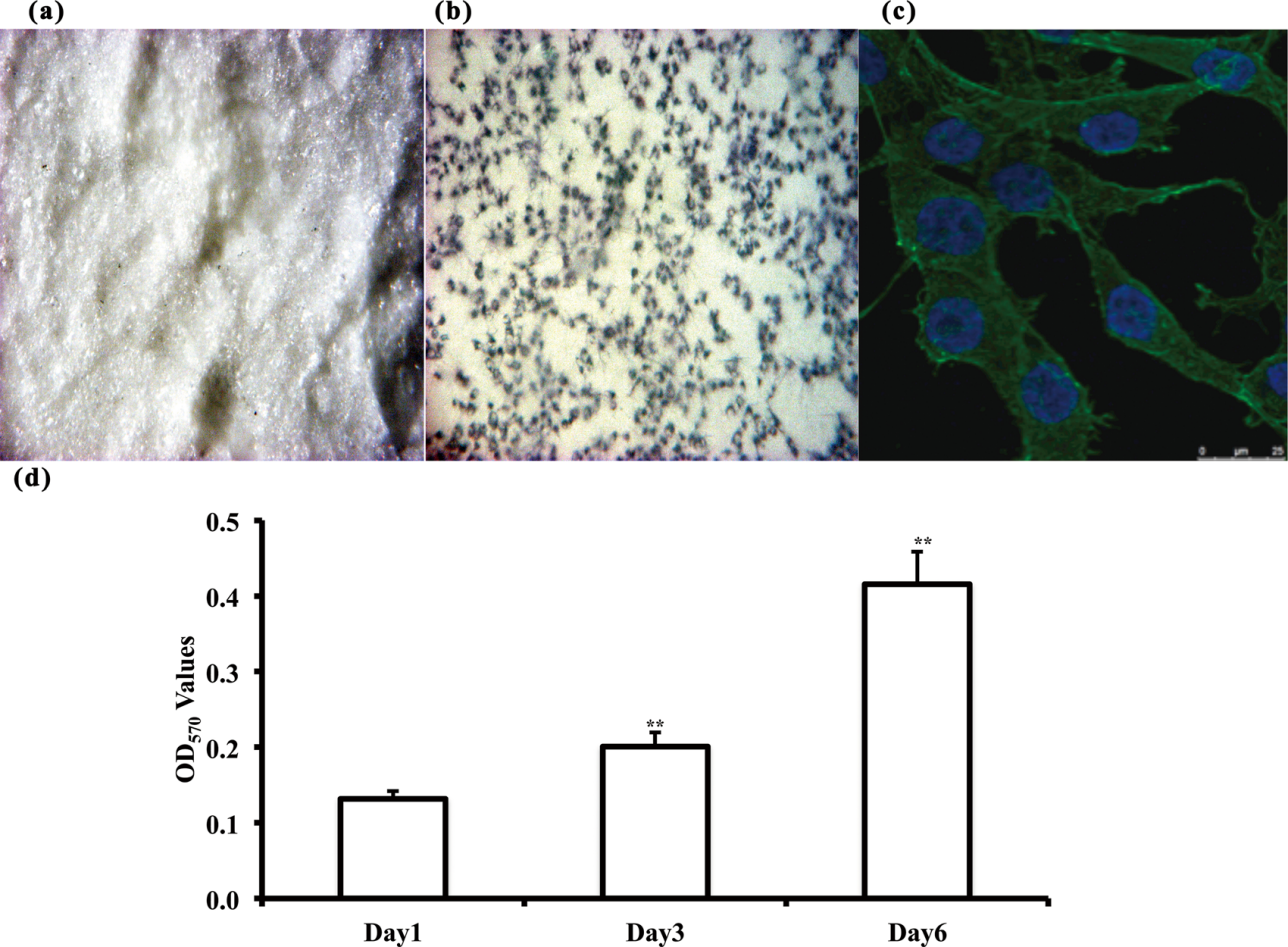
Osteoblast-like MG-63 cells attach on the CS5 scaffold. The high affinity of human bone cells indicated that the fabricated biocomposites act as a biomimic of the human bone scaffold. Live color staining and fluorescence graphs of Nuclear/Actin contact analysis are shown in upper panel. (a) unattached scaffold, (b) live cell staining of formazan on scaffold, and (c) fluorescence on scaffold. The live cells on scaffolds were incubated in a tetrazolium dye bath at 37°C for 4 h. Green signals indicate actin, and blue signals indicate the nuclear site of cells. (d). CS5 scaffolds showing a long-term survival period of 1–6 days. Cell numbers corresponding to the OD_570_ values represented approximately 836 ± 37 cells for each 0.1 OD value by MTT assay, for normalization with cell counting. ** P<0.01 compared to day 1 group.

To further demonstrate bone cell attachment on the CS5 silica bioceramic bone scaffolds (Fig 10a), we used two methods. First, we attempted to grow cells directly on the scaffold surface for approximately 24 hours and observed the growth using live staining with MTT direct uptake as shown in Fig 10b. Second, the cell morphology of the scaffold is shown by molecular probe fluorescence staining in Fig 10c. We further evaluated MG-63 cells during a long setting period on CS5 scaffold. We incubated the MG-63 cells for 1, 3, and 6 days on CS5 scaffold. As shown in Fig 10d, the cells proliferated twofold and fourfold at day 3 and day 6, respectively. The data showed long-term cell survival on the bioceramic scaffold, meaning that the cells were adapted to the CS5 scaffold environment. Future experiments on real-time cell penetration on 3D scaffolds will allow further evaluation of the long-term cell growth and differentiation states in the scaffolds *in vitro* and *in vivo*.

## Conclusions

The LAG method and a home-made 3DP machine were used to produce bioceramic bone scaffold. The compressive strength was increased by adding 5% wt. CaCO_3_ to SiO_2_ slurry (CS5) sintering at 1300°C. The bone scaffold of CS5 could precipitate needle-like β-CaSiO_3_ crystals after heat treatment. The maximum compressive strength of CS5 was 47 MPa and the porosity was increased to 34%. The optimum CS5 scaffold shows no cytotoxicity and good bone cell attachment and growth. The inter-porous silica bioceramic scaffolds with a pore size of 0.8 mm has been successfully fabricated. All of these results indicate that 3D artificial bone scaffolds of CS5 are suitable for medical implantation, have biomimetic strength-porosity compared to human bone, and can be expected to debut in the bone repair market in the near future.
